# Stem Cell Mobilization with G-CSF versus Cyclophosphamide plus G-CSF in Mexican Children

**DOI:** 10.1155/2016/4078215

**Published:** 2016-01-06

**Authors:** José Eugenio Vázquez Meraz, José Arellano-Galindo, Armando Martínez Avalos, Emma Mendoza-García, Elva Jiménez-Hernández

**Affiliations:** ^1^Banco de Sangre, Hospital General de Ecatepec las Américas, Simón Bolivar esq. Libertadores de América Fracc. las Américas, 55076 Ecatepec de Morelos, MEX, Mexico; ^2^Area de Virología, Laboratorio de Microbiología y Enfermedades Infecciosas, Hospital Infantil de México Federico Gómez, Mexico; ^3^Departamento de Oncología, Instituto Nacional de Pediatría, Mexico; ^4^Laboratorio de Hematología e Investigación, Hospital General de México, OD and Laboratorio Clínico, Central Hospital Infantil de México Federico Gómez, Ciudad de México, DF, Mexico; ^5^Departamento de Hematología Pediátrica, UMAE CMN la Raza IMSS, Mexico

## Abstract

Fifty-six aphaereses were performed in 23 pediatric patients with malignant hematological and solid tumors, following three different protocols for PBPC mobilization and distributed as follows: A: seventeen mobilized with 4 g/m^2^ of cyclophosphamide (CFA) and 10 *μ*g/kg/day of granulocyte colony stimulating factor (G-CSF), B: nineteen with CFA + G-CSF, and C: twenty only with G-CSF when the WBC count exceeded 10 × 10^9^/L. The average number of MNC/kg body weight (BW)/aphaeresis was 0.4 × 10^8^ (0.1–1.4), 2.25 × 10^8^ (0.56–6.28), and 1.02 × 10^8^ (0.34–2.5) whereas the average number of CD34+ cells/kg BW/aphaeresis was 0.18 × 10^6^/kg (0.09–0.34), 1.04 × 10^6^ (0.19–9.3), and 0.59 × 10^6^ (0.17–0.87) and the count of CFU/kg BW/aphaeresis was 1.11 × 10^5^ (0.31–2.12), 1.16 × 10^5^ (0.64–2.97), and 1.12 × 10^5^ (0.3–6.63) in groups A, B, and C, respectively. The collection was better in group B versus group A (*p* = 0.007 and *p* = 0.05, resp.) and in group C versus group A (*p* = 0.08 and *p* = 0.05, resp.). The collection of PBPCs was more effective in the group mobilized with CFM + G-CSF when the WBC exceeded 10 × 10^3^/*μ*L in terms of MNC and CD34+ cells and there was no toxicity of the chemotherapy.

## 1. Introduction

With the discovery that peripheral blood progenitor cells (PBPC) could be obtained by aphaeresis, several reports have shown that these stem cells can be used to reconstitute hematopoiesis, after myeloablative therapy in cancer patients [1–3]. Chemotherapy increases the amount of PBPC 20–50 times [4, 5]. Therefore high dose of cyclophosphamide (CFA) has been frequently used to mobilize PBPC [6–10]. Hematopoietic growth factors such as G-CSF and GM-CSF used after chemotherapy increase the efficacy of stem cells mobilization even more. However, G-CSF in combination with chemotherapy must be administrated during 8–12 days compared with only 4 to 6 days when it is applied without chemotherapy [11–16].

There is not enough experience in children to establish the optimal method for PBPC mobilization, as it could be done with hematopoietic growth factors either alone or in combination with chemotherapy. We determined the number of mononuclear cells (MNC), CD34+ cells, and colony forming units (CFU) in the leukapheresis products of pediatric patients with malignant hematological diseases and solid tumors, following three different protocols for stem cells mobilization.

## 2. Patients and Methods

The study included twenty-three pediatric patients with malignant hematological diseases and solid tumors. The main characteristics of the patients are shown in Table 1. Parent's consent of the study was obtained in all cases.

### 2.1. PBPC Mobilization

The patients were divided into three groups: group A was assigned to high dose (4 g/m^2^) of cyclophosphamide (CFA) and (10 *μ*g/kg/day) of G-CSF applied subcutaneously, and the aphaeresis procedures were started when the white blood cell count (WBC) exceeded 1.0 × 10^9^/L. Group B was subjected to the same regimen (CFM + G-CSF), but the WBC was >10 × 10^9^/L at the time of starting the cell collection,and group C was treated subcutaneously with G-CSF alone for 4 days and the aphaeresis was started at day 5.

### 2.2. PBPC Collection

Collections were performed by placement of a double lumen dialysis catheter with a Baxter Fenwal CS 3000 plus machine using large volume leukapheresis (LVL) (200 mL/kg). The inlet flow was 30–50 mL/min. The target number of MNC and CD34+ cells was 4 × 10^8^/kg and 2 × 10^8^/kg, respectively. A minimum of 1 × 10^6^/kg CD34+ cells and 2 × 10^8^/kg of MNC were considered sufficient though. When the yield of a single aphaeresis was considered insufficient, the process was continued daily until the CD34+ cells and MNC target dose were achieved. The final products were frozen and stored in liquid nitrogen at −196°C.

### 2.3. Aphaeresis Products

The obtained product of MNC was processed by Coulter Max M, and the number of CD34+ cells was determined by flow cytometry in a FACSCalibur by using the ProCount software (Becton Dickinson).

### 2.4. Cell Cultures

The cell cultures were prepared in Methocult GF H4434 (Stem Cell Technology Inc, Vancouver, BC), contained 1 × 10^5^ MNCs per mL, and were incubated at 37°C, in presence of CO_2_, and the colony forming units (CFUs) were determined on day 14 by using an inverted microscope as described previously [3].

### 2.5. Statistical Analysis

Not normally distributed data are presented as median and range. Differences were compared using the nonparametrical Kruskal-Wallis test. The Stata software program (Stata Corporation, College Station, TX, USA) was used for statistical analysis.

## 3. Results

### 3.1. Patient's Characteristics

Twenty-three children were included in the study. Seven patients were assigned to group A, eight to group B, and eight to group C. Seventeen, nineteen, and twenty leukaphereses were performed in each group, respectively. Patients characteristics are shown in Table 1.

The average number of days of G-CSF administration was 6.1 (4–8) in group A, 11.8 (10–15) in group B, and 5.7 (5–7) in group C. The average of MNC in the aphaeresis products was 0.4 × 10^8^/kg (0.1–1.4) in group A, 2.25 × 10^8^ (0.56–6.28) in group B, and 1.02 × 10^8^ (0.34–2.5) in group C. The mean number of CD34+ cells was 0.18 × 10^6^/kg BW (0.09–0.34) in group A, 1.04 × 10^6^ (0.19–9.3) in group B, and 0.59 × 10^6^ (0.17–0.87) in group C. The mean count of CFU/kg BW was 1.11 × 10^5^ (0.31–2.12) in group A, 1.16 × 10^5^ (0.64–2.97) in group B, and 1.12 × 10^5^ (0.3–6.63) in group C (Table 2).

The differences between the three groups were statistically significant for the number of MNC/kg BW (*p* = 0.007) and CD34+ cells/kg (*p* = 0.05) in group B versus group A and for CD34+ cells in the group C versus group A (*p* = 0.05). The UFC × 10^5^/kg BW was similar in the different groups (Table 3).

In the group treated with chemotherapy, patients were hospitalized due to severe neutropenia (<0.5 × 10^9^/L), but no one required antibiotic or platelet transfusion.

## 4. Discussion

Mobilized peripheral blood is now the main hematopoietic progenitor cell source for cellular support following myeloablative chemotherapy. PBPC transplantation results in a more rapid hematopoietic recovery than bone marrow cell transplantation, mainly due to the larger number of hematopoietic progenitor cells infused. G-CSF alone or combined with chemotherapy is commonly used in mobilization protocols and it appears to be able to achieve adequate progenitor cell yields for single transplants in most patients and [17] observed that the combination of myelosuppressive chemotherapy and G-CSF can mobilize better PBPC than chemotherapy alone and the administration of these cytokines in combination with chemotherapy may reduce the blood volume processed by leukapheresis. Additionally the optimal procedure for PBPC mobilization in children has not yet been determined because data on the engraftment are not enough [17–19]. Clinical collection of PBPC by leukapheresis has been more difficult to perform in a child than in an adult, especially after receiving an extensive therapy for their malignancies [20–22]. It has been shown that heavy and prolonged cytotoxic treatments seem to exhaust the mobilizable stem cell pool; therefore PBPC should be collected as early as possible after diagnosis [17, 23].

In order to optimize mobilization and to obtain a larger number of CD34+ cells, we simultaneously administered CFA + G-CSF or only G-CSF. Our results demonstrated that the number of CD34+ cells collected was similar for all groups (Table 2). The difference in obtained results was statistically significant when we compared group B (CFA + G-CSF WBC > 10 × 10^3^/*μ*L) and group A (CFA + G-CSF WBC > 1.0 × 10^3^/*μ*L) (*p* = 0.05) and also for group C (G-CSF) and group A (*p* = 0.05), but it was not statistically significant for group B versus group C. This suggests that the efficacy of G-CSF is similar in the presence or absence of CFA, when the leucocytes count exceeded 10 × 10^9^/L; findings similar to ours were reported by Costa et al. [17]. The difference in the amount of UFC was not statistically significant between the studied groups.

The main limitations of chemotherapy mobilizations are neutropenia, sepsis, and bleeding diathesis and the unpredictability of starting time for collection procedure [24–27]. In our group of patients with CFA, there have been no serious complications, but patients did require more days of G-CSF application.

From the present data, we conclude that PBPC mobilization with CFM + G-CSF when the WBC is >10 × 10^9^/L has a similar efficacy in comparison with mobilization using only G-CSF, and the last one has less adverse effects.

## Figures and Tables

**Table 1:** Clinical characteristics of the patients.

Number	Age (years)	Sex	Weight (Kg.)	Diagnosis	
1	3	M	15	WT S-IV	1°CR
2	10	F	39	LMA-M2	2°CR
3	16	M	38	HD S-IVB	2°CR
4	7	F	22	LMA-M1	1°CR
5	3	F	15	LAL-L1 t(9,22)	1°CR
6	3	M	17	LAL-L1 t(4:11)	1°CR
7	9	F	32	LAL-L1 t(9,22)	1°CR
8	12	M	32	HD S-IVB	2°CR
9	16	M	55	LAL-L2 t(9,22)	1°CR
10	11	M	56	LMA-M4	1°CR
11	16	M	43	LAL-L1 t(9:22)	2°CR
12	2	M	13	NBL S-IV	3°CR
13	8	F	29	LAL-L2 t(9,22)	1°CR
14	8	M	26	LMA-M4	1°CR
15	9	F	22	WT S-IV	3°CR
16	4	M	21	LAL-L1 t(9,22)	1°CR
17	6	M	25	LMA-M2	2°CR
18	5	F	22	WT S-IV	2°CR
19	7	F	21	LMA-M1	2°CR
20	10	M	38	LAL-L1 t(9,22)	1°CR
21	8	M	25	LAL-L1 t(9,22)	1°CR
22	13	M	31	HD S-IVB	2°CR
23	6	F	26	LAL-L1 t(9,22)	1°CR

HD: Hodgkin disease, WT: Wilms' tumor, LMA: myeloblastic acute leukemia, LAL: lymphoblastic acute leukemia, NBL: neuroblastoma, CR: complete remission, and S: stage.

**Table 2:** PBPC results by group.

	Group A CFA + G-CSF WBC > 1.0 × 10^9^/L Mean and range	Group B CFA + G-CSF WBC > 10 × 10^9^/L Mean and range	Group C G-CSF alone Mean and range
Days of G-CSF	6.1 (4–8)	11.8 (10–15)	5.7 (5–7)
MNC **× **10^8^/kg	0.4 (0.1–1.4)	2.25 (0.56–6.28)	1.02 (0.34–2.5)
CD34+ **× **10^6^/kg	0.18 (0.09–0.34)	1.04 (0.19–9.3)	0.59 (0.17–0.87)
CFU **× **10^5^/kg	1.11 (0.31–2.12)	1.16 (0.64–2.97)	1.12 (0.3–6.63)

WBC: white blood cells; MNC: mononuclear cells; CFU: colony forming units.

**Table 3:** Statistical analysis using the nonparametrical Kruskal-Wallis test.

	MNC × 10^8^/kg	CD34+ cells × 10^6^/kg	UFC × 10^5^/kg
A versus B	0.007	0.055	0.297
A versus C	0.082	0.055	0.526
B versus C	0.248	0.172	0.833
