# Corrigendum to “Stem Cell Mobilization with G-CSF versus Cyclophosphamide plus G-CSF in Mexican Children”

**DOI:** 10.1155/2016/3914030

**Published:** 2016-10-03

**Authors:** José Eugenio Vázquez Meraz, José Arellano-Galindo, Armando Martínez Avalos, Emma Mendoza-García, Elva Jiménez-Hernández

**Affiliations:** ^1^Banco de Sangre, Hospital General de Ecatepec las Américas, Simón Bolivar esq. Libertadores de América Fracc. las Américas, 55076 Ecatepec de Morelos, MEX, Mexico; ^2^Area de Virología, Laboratorio de Microbiología y Enfermedades Infecciosas, Hospital Infantil de México Federico Gómez, Mexico; ^3^Departamento de Oncología, Instituto Nacional de Pediatría, Mexico; ^4^Laboratorio de Hematología e Investigación, Hospital General de México, OD and Laboratorio Clínico, Central Hospital Infantil de México Federico Gómez, Ciudad de México, DF, Mexico; ^5^Departamento de Hematología Pediátrica, UMAE CMN la Raza IMSS, Mexico

In the article titled “Stem Cell Mobilization with G-CSF versus Cyclophosphamide plus G-CSF in Mexican Children” [1], there were some typos which should be corrected as follows.

UFC should be corrected to CFU in the third paragraph of the Results, Table 3, and the last paragraph of the Discussion.

CFM should be corrected to CFA in the Abstract and the last paragraph of the Discussion.

LAL should be corrected to ALL in Table 1.

## References

[1] Meraz J. E. V., Arellano-Galindo J., Avalos A. M., Mendoza-García E., Jiménez-Hernández E. (2016). Stem cell mobilization with g-csf versus cyclophosphamide plus g-csf in mexican children. *Stem Cells International*.

